# Lack of Significant Association between Plasma/Serum miR-221 Expression and Poor Survival of Carcinoma: A Meta-Analysis

**DOI:** 10.1155/2013/394030

**Published:** 2013-11-04

**Authors:** Min-hua Rong, Yi-wu Dang, Gang Chen

**Affiliations:** ^1^Research Department, Affiliated Cancer Hospital, Guangxi Medical University, 71 Hedi Road, Nanning, Guangxi Zhuang Autonomous Region 530021, China; ^2^Department of Pathology, First Affiliated Hospital, Guangxi Medical University, 6 Shuangyong Road, Nanning, Guangxi Zhuang Autonomous Region 530021, China

## Abstract

*Background*. MicroRNAs (miRNAs) exhibit altered expression levels in cancers, and they may play a potential role as diagnostic and prognostic biomarkers of cancers. The aim of this meta-analysis was to summarize recent advances in miR-221 involvement in a variety of carcinomas and derive a more precise estimation of the relationship between circulating miR-221 level and survival of cancer patients. *Methods*. We searched online PubMed, EMBASE, and Cochrane Library up to August 2013 to identify relevant studies. Data were collected from studies comparing survival in patients with various carcinomas with higher miR-221 expression to those with lower levels. Pooled hazard ratios (HRs) of miR-221 for survival were calculated. *Results*. There were 4 studies included in the meta-analysis. The results of meta-analysis suggested that no significant difference in poor overall survival between miR-221 high and low groups (OR = 0.94, 95%, CI = 0.47–1.87, *Z* = 0.17, and *P* = 0.863). *Conclusions*. The current meta-analysis showed the equivalence of high and low plasma/serum miR-221 expression for carcinomas in terms of survival.

## 1. Introduction

In recent years, microRNAs (miRNAs) have received great attention in cancer research. MiRNAs are small noncoding RNAs, usually 20–23 nucleotide (nt) long, which regulate the expression of protein-coding genes at the posttranscriptional level. Studies have also shown that aberrant miRNA expression is involved in the development and progression of cancer [1–3]; thus miRNAs could be used as biomarkers for diagnosis and prognosis of cancer, and targets for cancer molecular therapy [4–6]. Nevertheless, there is still a lot remaining to be understood in the involvement of miRNAs in carcinogenesis and progression of cancer. Among all the cancer-related miRNAs, miR-221 was reported to be increasingly expressed in various carcinomas, compared with nontumoral tissues [7–9]. However, as a noninvasive method, detection of the circulating miRNA biomarkers in plasma or serum samples is more acceptable than those in tissue specimens.

To assess the cumulative evidence regarding the possible association between elevated circulating miR-221 and poor survival in patients with cancer and to discuss the possibility to apply miR-221 as a prognostic marker, we conducted a systematic review and meta-analysis of relevant studies investigating this association.

## 2. Methods

### 2.1. Data Sources

The study was performed following the guidelines of the Meta-analysis of Observational Studies in Epidemiology group (MOOSE) [10]. We carefully searched online PubMed, EMBASE, and Cochrane Library up to August 2013 to identify relevant studies. Two sets of keywords used in this search were “(miR-221 and cancer) or (miRNAs and cancer prognosis)” with limits to article types other than review, human species, and English language. 

### 2.2. Study Selection

All identified studies were examined by 2 authors (M-H Rong and G.Chen) independently. Studies were considered eligible if they met the following criteria: (1) they were published in a peer-reviewed journal; (2) they studied the patients with any type of carcinoma; (3) they reported miR-221 expression in blood, plasma, or serum; and (4) they investigated the association between miR-221 expression levels and survival outcome. The exclusion criteria for the current meta-analysis were that studies (1) reporting the survival data of a set of miRNAs but not miR-221 alone, (2) analyzing nondichotomous miR-221 expression levels, (3) without key information such as hazard ratio (HR), 95%* *CI and *P* value, and (4) were laboratory studies. 

### 2.3. Data Extraction

The following information from each paper was extracted by an author (M-H Rong) and then confirmed by another author (G. Chen): first author, publish year, country of publication, type of carcinoma, clinical stage of disease, number of patients, specimen, measurement and cutoff of miR-221, survival analysis, HR, 95% CI and *P* value, and time of followup. These extracted data were double checked by Y-W Dang. Additionally, we emailed to the authors of studies for the data needed for the meta-analytic calculations.

### 2.4. Statistical Analysis

Statistics were conducted by the Stata 11.0 statistical software. The random effect model was used to calculate the pooled HR and its 95% CI. The heterogeneity test of combined HR was evaluated via the *I*-square test and Chi-square. A two-tailed *P* value of <0.05 was considered statistically significant.

## 3. Results

### 3.1. Four Hundred and Forty-Eight Cases Were Involved in Meta-Analysis

**Figure 1:**
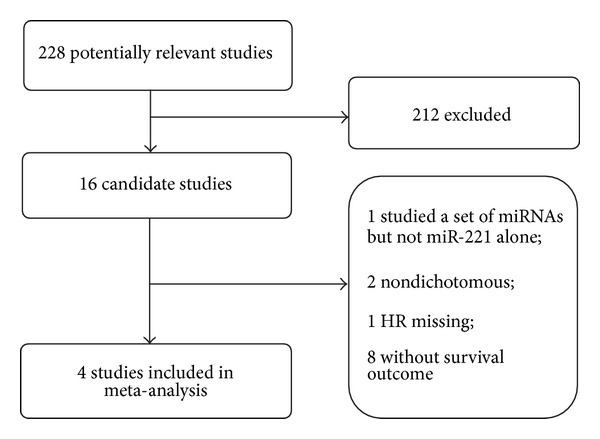
Flow diagram of search strategy.

Two hundred and twenty-eight records for miR-221 and cancer were identified from a primary literature search in PubMed, EMBASE, and Cochrane Library. After manually screening the titles, abstracts, and key words, two hundred and twelve studies were excluded because they were review articles, letters, non-English articles, laboratory studies, or studies irrelevant to the current analysis. Of the sixteen candidate studies, one study investigated a set of miRNAs but not miR-221 alone; two studies did not deal with miR-221 expression data as a dichotomic variable; one lacked the key HR data; and other eight had no survival outcome. Therefore, the final meta-analysis was carried out for the remaining four studies [11–14] (Figure 1). The main features of eligible studies are summarized in Table 1. We collected data from 4 studies including a total of 448 participants from the United States and China. All of them were retrospective in design. The patients were of four types of carcinomas, including colorectal cancer (CRC) [11], nonsmall cell lung carcinoma (NSCLC) [12], hepatocellular carcinoma (HCC) [13], and NK/T-cell lymphoma (NTCL) [14]. Quantitative real-time PCR (qRT-PCR) was applied for miR-221 expression assessment in all the studies included. Plasma or serum samples were examined to determine miR-221 expression level, while both the two samples were tested in one study [12]. In addition, the cut-off values of miR-221 were based on Youden index (two studies), median (one study), or 4.8-fold (one study). Overall survival (OS) was evaluated in all the studies.

### 3.2. Lack of Significant Association between miR-221 Expression and Poor Overall Survival

**Figure 2:**
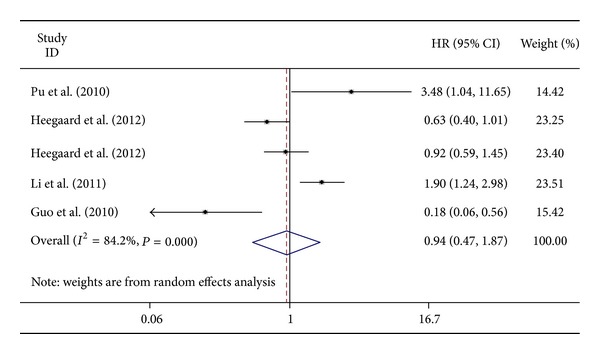
Forrest plots of overall survival in cancer patients evaluating hazard ratios of high miR-221 expression as compared to low expression. Diamonds indicate pooled OR in single study; horizontal bars indicate 95% CI.

There was significant heterogeneity among studies (*x* ^2^ for heterogeneity = 25.32, *I*-square = 84.2%, *P* = 0.000, and Freedom = 4). The results of meta-analysis suggested that no significant difference in poor overall survival between miR-221 high and low groups (OR = 0.94, 95% CI = 0.47–1.87, *Z* = 0.17, and *P* = 0.863) (Figure 2). Since there were less than five prognostic studies in the current analysis, publication bias of the studies included was not performed.

## 4. Discussion

Extensive profiling studies over the past several years have shown that various miRNAs are differentially expressed in different classes of cancers. Among them, miR-221 is considered as a microoncogene. MiR-221 plays an important role in epithelial-to-mesenchymal transition (EMT). It has been identified as a basal-like subtype-specific miRNA that downregulates the expression of epithelial-specific genes and enhances the expression of mesenchymal-specific genes. Furthermore, miR-221 increases cell migration and invasion [15–17]. The basal-like transcription factor, FOSL1, can directly stimulate the transcription of miR-221 [17]. The abundance of miR-221 reduces with the suppression of mitogen-activated or extracellular signal-regulated protein kinase (MEK) [17]. The miR-221-mediated reduction in E-cadherin is dependent on the targeting of the 3′-UTR of trichorhinophalangeal syndrome type 1 (TRPS1). TRPS1 inhibits EMT by directly repressing the expression of Zinc finger E-box-binding homeobox 2 (ZEB2) [18]. Thus, miR-221 could contribute to the aggressive clinical behavior of various types of cancers.

Other crucial validated miR-221 target genes include an oncosuppressor p27^Kip1^ and a key transcription factor, Slug. Interference with the process of Slug/miR-221 upregulation and p27^Kip1^ downregulation can be accomplished using antisense miRNA (antagomiR or miRNA inhibitor) molecules targeting miR-221, inducing the downregulation of Slug and the upregulation of p27^Kip1^ [19–21]. This may provide new therapeutic options for cancers when systematic delivery of anti-miR-221 is achievable.

Overexpression of miR-221 has been confirmed in several malignancies, including HCC [22], breast cancer [23], prostate carcinoma [24], colorectal carcinoma [25], melanoma [26], and acute myeloid leukemia [27]. High level of miR-221 expression is correlated with metastasis, tumor capsular infiltration, tumor stage [22, 28, 29], and poor prognosis [30, 31]. Although many miRNAs are expressed in tumor tissues and tumor cells, the development of such miRNAs being biomarkers requires a more convenient approach of studying peripheral blood, rather than tissue collection. Circulating prognostic markers should be more valuable for detection throughout life of patients with carcinoma. Some miRNAs were recently identified in serum and plasma in a remarkably stable form that is protected from endogenous RNase activity [6, 32]. The current study aims to provide a comprehensive evaluation on the association between serum/plasma of miR-221 expression and survival of carcinomas based on published references available. However, the results of this meta-analysis demonstrate that there is no significant difference in poor overall survival (OR = 0.94, 95% CI = 0.47–1.87, *Z* = 0.17, and *P* = 0.863) between high and low miR-221 in patients with carcinoma. It is unlikely that serum/plasma miR-221 overexpression is an independent prognostic factor for tumor prognosis.

However, this review has heterogeneity and several limitations. First, the number of studies included in the study is relatively small. The pooled HR was calculated on the basis of 4 studies with a small sample size of 448 cases. Second, many factors including cut-off definition of miR-221 and tumor clinical stage which may influence the tumor progression are not considered in the meta-analysis. Third, we did not consider sample size in trials, which can vary in the studies included. Smaller number in trials might give different results compared to larger trials for disease progression. For instance, the number of the cases in the study of Heegaard et al. [12] was approximately 5 times that in the study of Li et al. [13]. The publication bias was not excluded either. Another potential limitation of this study is that there was marked heterogeneity in the modes of treatment used in each study and response rates. Considerable between-study heterogeneity was observed probably due to the reasons listed above.

Despite these caveats, this meta-analysis demonstrates the equivalence of high and low miR-221 expression for carcinomas in terms of survival.

## 5. Conclusions

In conclusion, a causal effect of plasma/serum miR-221 expression on tumor prognosis is unlikely, given the apparent lack of an association based on current small numbers of studies and patients. Future studies with a large number of cases are recommended to further validate the role of circulating miR-221 as the prognostic indicator for patients with carcinoma.

## Figures and Tables

**Table 1:** Summary information of references included in the meta-analysis of miR-221 and survival.

Author	Year	Country	Study design	Type of carcinoma	Stage	Number of samples	Specimen	miR-221 assay	Cutoff	Survival analysis	Followup, months	Hazard ratios	CI	*P*
Pu et al. [11]	2010	China	R	CRC	I–IV	103	Plasma	qRT-PCR	Youden index	OS	60	Reported 3.48	1.04–11.65	0.043
Heegaard et al. [12]	2012	USA	R	NSCLC	I-II	220	Serum	qRT-PCR	median	OS	60	AP 0.63	0.40–1.01	0.053
Heegaard et al. [12]	2012	USA	R	NSCLC	I-II	220	Plasma	qRT-PCR	median	OS	60	AP 0.92	0.59–1.45	0.723
Li et al. [13]	2011	China	R	HCC	I–IV	46	Serum	qRT-PCR	4.8-fold	OS	60	Reported 1.90	1.24–2.98	0.018
Guo et al. [14]	2010	China	R	NTCL	I–IV	79	Plasma	qRT-PCR	Youden index	OS	60	Reported 0.18	0.06–0.56	0.003

R: retrospective; CRC: colorectal cancer; NSCLC: nonsmall cell lung carcinoma; HCC: hepatocellular carcinoma; NTCL: NK/T-cell lymphoma; OS: overall survival; AP: author provided.

## References

[1] Ma L, Weinberg RA (2008). MicroRNAs in malignant progression. *Cell Cycle*.

[2] Bullock MD, Sayan AE, Packham GK, Mirnezami AH (2012). MicroRNAs: critical regulators of epithelial to mesenchymal (EMT) and mesenchymal to epithelial transition (MET) in cancer progression. *Biology of the Cell*.

[3] Fang YX, Gao WQ (2013). Roles of microRNAs during prostatic tumorigenesis and tumor progression. *Oncogene*.

[4] Tousoulis D (2013). Novel biomarkers in the prognosis, progression and treatment of cardiovascular disease: the role of microRNAs. *Current Topics in Medicinal Chemistry*.

[5] Dickinson BA, Semus HM, Montgomery RL (2013). Plasma microRNAs serve as biomarkers of therapeutic efficacy and disease progression in hypertension-induced heart failure. *European Journal of Heart Failure*.

[6] Brase JC, Wuttig D, Kuner R, Sültmann H (2010). Serum microRNAs as non-invasive biomarkers for cancer. *Molecular Cancer*.

[7] Pineau P, Volinia S, McJunkin K (2010). miR-221 overexpression contributes to liver tumorigenesis. *Proceedings of the National Academy of Sciences of the United States of America*.

[8] Kawaguchi T, Komatsu S, Ichikawa D (2013). Clinical impact of circulating miR-221 in plasma of patients with pancreatic cancer. *British Journal of Cancer*.

[9] Garofalo M, Di Leva G, Romano G (2009). miR-221&222 regulate TRAIL resistance and enhance tumorigenicity through PTEN and TIMP3 downregulation. *Cancer Cell*.

[10] Stroup DF, Berlin JA, Morton SC (2000). Meta-analysis of observational studies in epidemiology: a proposal for reporting. *Journal of the American Medical Association*.

[11] Pu XX, Huang GL, Guo HQ (2010). Circulating miR-221 directly amplified from plasma is a potential diagnostic and prognostic marker of colorectal cancer and is correlated with p53 expression. *Journal of Gastroenterology and Hepatology*.

[12] Heegaard NH, Schetter AJ, Welsh JA, Yoneda M, Bowman ED, Harris CC (2012). Circulating micro-RNA expression profiles in early stage nonsmall cell lung cancer. *International Journal of Cancer*.

[13] Li J, Wang Y, Yu W, Chen J, Luo J (2011). Expression of serum miR-221 in human hepatocellular carcinoma and its prognostic significance. *Biochemical and Biophysical Research Communications*.

[14] Guo HQ, Huang GL, Guo CC, Pu X, Lin T (2010). Diagnostic and prognostic value of circulating miR-221 for extranodal natural killer/T-cell lymphoma. *Disease Markers*.

[15] Howe EN, Cochrane DR, Richer JK (2012). The miR-200 and miR-221/222 microRNA families: opposing effects on epithelial identity. *Journal of Mammary Gland Biology and Neoplasia*.

[16] Hwang MS, Yu N, Stinson SY (2013). miR-221/222 targets adiponectin receptor 1 to promote the epithelial-to-mesenchymal transition in breast cancer. *PLoS One*.

[17] Stinson S, Lackner MR, Adai AT (2011). TRPS1 targeting by miR-221/222 promotes the epithelial-to-mesenchymal transition in breast cancer. *Science Signaling*.

[18] Stinson S, Lackner MR, Adai AT (2011). miR-221/222 targeting of trichorhinophalangeal 1 (TRPS1) promotes epithelial-to-mesenchymal transition in breast cancer. *Science Signaling*.

[19] Piva R, Spandidos DA, Gambari R (2013). From microRNA functions to microRNA therapeutics: novel targets and novel drugs in breast cancer research and treatment (Review). *International Journal of Oncology*.

[20] Lambertini E, Lolli A, Vezzali F, Penolazzi L, Gambari R, Piva R (2012). Correlation between Slug transcription factor and miR-221 in MDA-MB-231 breast cancer cells. *BMC Cancer*.

[21] Nassirpour R, Mehta PP, Baxi SM, Yin MJ (2013). miR-221 promotes tumorigenesis in human triple negative breast cancer cells. *PLoS One*.

[22] Rong M, Chen G, Dang Y (2013). Increased MiR-221 expression in hepatocellular carcinoma tissues and its role in enhancing cell growth and inhibiting apoptosis in vitro. *BMC Cancer*.

[23] Hui AB, Shi W, Boutros PC (2009). Robust global micro-RNA profiling with formalin-fixed paraffin-embedded breast cancer tissues. *Laboratory Investigation*.

[24] Zheng C, Yinghao S, Li J (2012). MiR-221 expression affects invasion potential of human prostate carcinoma cell lines by targeting DVL2. *Medical Oncology*.

[25] Sun K, Wang W, Zeng J, Wu C, Lei S, Li G (2011). MicroRNA-221 inhibits CDKN1C/p57 expression in human colorectal carcinoma. *Acta Pharmacologica Sinica*.

[26] Felicetti F, Errico MC, Bottero L (2008). The promyelocytic leukemia zinc finger-microRNA-221/-222 pathway controls melanoma progression through multiple oncogenic mechanisms. *Cancer Research*.

[27] Cammarata G, Augugliaro L, Salemi D (2010). Differential expression of specific microRNA and their targets in acute myeloid leukemia. *American Journal of Hematology*.

[28] Veerla S, Lindgren D, Kvist A (2009). MiRNA expression in urothelial carcinomas: important roles of miR-10a, miR-222, miR-125b, miR-7 and miR-452 for tumor stage and metastasis, and frequent homozygous losses of miR-31. *International Journal of Cancer*.

[29] Lu X, Zhao P, Zhang C (2009). Analysis of miR-221 and p27 expression in human gliomas. *Molecular Medicine Reports*.

[30] Radojicic J, Zaravinos A, Vrekoussis T, Kafousi M, Spandidos DA, Stathopoulos EN (2011). MicroRNA expression analysis in triple-negative (ER, PR and Her2/neu) breast cancer. *Cell Cycle*.

[31] Hong F, Li Y, Xu Y, Zhu L (2013). Prognostic significance of serum microRNA-221 expression in human epithelial ovarian cancer. *The Journal of International Medical Research*.

[32] Chen X, Ba Y, Ma L (2008). Characterization of microRNAs in serum: a novel class of biomarkers for diagnosis of cancer and other diseases. *Cell Research*.

